# Determination of Novel Highly Effective Necrostatin Nec-1s in Rat Plasma by High Performance Liquid Chromatography Hyphenated with Quadrupole-Time-of-Flight Mass Spectrometry

**DOI:** 10.3390/molecules23081946

**Published:** 2018-08-03

**Authors:** Peter Mikuš, Daniel Pecher, Drahomíra Rauová, Csaba Horváth, Adrián Szobi, Adriana Adameová

**Affiliations:** 1 Department of Pharmaceutical Analysis and Nuclear Pharmacy, Faculty of Pharmacy, Comenius University in Bratislava, Odbojárov 10, 832 32 Bratislava, Slovak Republic; pecher1@uniba.sk (D.P.); rauova@fpharm.uniba.sk (D.R.); 2 Toxicological and Antidoping Center, Faculty of Pharmacy, Comenius University in Bratislava, Odbojárov 10, 832 32 Bratislava, Slovak Republic; 3 Department of Pharmacology and Toxicology, Faculty of Pharmacy, Comenius University in Bratislava, Odbojárov 10, 832 32 Bratislava, Slovak Republic; csaba125@gmail.com (C.H.); adrian.szobi@gmail.com (A.S.); adameova@fpharm.uniba.sk (A.A.)

**Keywords:** high performance liquid chromatography, quadrupole-time-of-flight mass spectrometry, necrostatin Nec-1s, rat plasma, determination, biomedical analysis

## Abstract

Necrostatins have been shown to retard necroptosis, a programmed necrotic-like cell death, which has been shown to underlie pathophysiology of various diseases. Nec-1s, a novel highly effective necrostatin, overcomes some drawbacks of former necrostatin analogues. The determination of Nec-1s in biological system, however, has not been carried out so far. Therefore, this study was undertaken to optimize and validate the HPLC-DAD-Q-TOF method for the assessment of Nec-1s levels in the plasma what is the necessity for designing its proper dosing regimen for in vivo studies. Benefits of the proposed analytical protocol include: (i) simple sample preparation (precipitation of plasma proteins, evaporation of acetonitrile, reconstitution in mobile phase), (ii) fast, selective and sensitive analysis due to a highly orthogonal LC-MS system providing less than 8 min analysis time, (iii) detection of Nec-1s without any matrix interferences, and quantitation of very low concentration levels of Nec-1s (LLOQ ~ 20 ng/mL), (iv) high reliability of Nec-1s determination with precision and accuracy values meeting the FDA criteria for biomedical analysis. The proposed analytical protocol is suitable for routine use in relevant biological studies, and, in this work, it was successfully applied for monitoring of Nec-1s plasma levels in rats providing reproducible and consistent results. Based on pharmacokinetic features, which can also be assessed due to the results of this study, there will be efforts to perform both acute and chronic in vivo studies and potential clinical safety studies first.

## 1. Introduction

**Figure 1:**
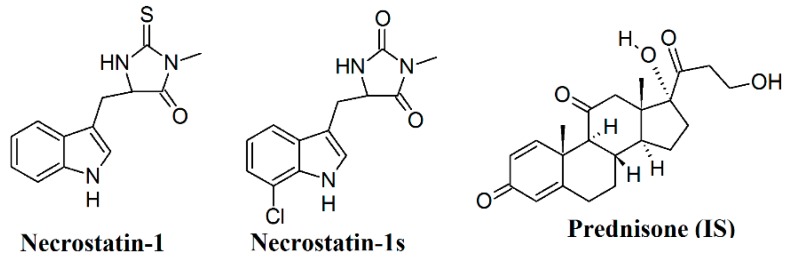
Chemical structures of necrostatin-1, necrostatin-1s, and prednisone (internal standard, IS).

Inhibition of receptor interacting protein 1 (RIP1) by a drug class referred to as the necrostatins has been shown to retard necroptosis, a form of programmed necrotic-like cell death which has been suggested to underlie pathophysiology of various diseases, including neurodegenerative diseases [1], acute inflammatory conditions [2], malignancies [3] and cardiovascular diseases associated with ischemia (reviewed by [4,5]). Necrostatin-1 (Nec-1, 5-(indol-3-ylmethyl)(2-thio-3-methyl)hydantoin), the first discovered necrostatin [6], which acts as an allosteric inhibitor of kinase domain of RIP1, has been widely used in some of these studies [4,7,8]. However, its relatively low kinase selectivity evidenced by the concomitant inhibition of indoleamine 2,3-dioxygenase (IDO), a potent immunomodulatory enzyme [9], may significantly influence the outcomes of such studies, mainly those associated with inflammatory response [10]. In addition, we have indicated that Nec-1, may also influence protein kinases regulating excitation-contraction coupling (ECC) of the heart and thereby its contractile and electrical activity [11]. An inactive analogue, Nec-1i (Nec-1i, N-demethylated thiohydantoin analogue of Nec-1), lacking the capability to inhibit RIP1 and probably also other protein kinases without any effects on ECC [11], can serve as a suitable control. However, this compound was also found to be an IDO inhibitor [10]. In spite of these limitations, other analogues of necrostatin-1 such as necrostatin-3,-5,-7, which differ in their mechanism of RIP1 inhibition, have not been shown to be superior to Nec-1 and are not routinely used. Instead, 5-((7-chloro-1*H*-indol-3-yl)methyl)-3-methyl-2,4-imidazolidinedione (Nec-1s), possesses several advantageous pharmacokinetic and pharmacodynamic characteristics in comparison to Nec-1. In fact, it is around two-fold more effective than Nec-1 (IC_50_ = 210 nM vs. 494 nM with Nec-1), does not influence IDO and is metabolically more stable (for the chemical structures of Nec-1 and Nec-1s, see Figure 1). Moreover, safety profile of Nec-1s is also superior to that of Nec-1; its in vivo and in vitro toxicity is reduced [10]. Another paper studied use of RIP1 inhibitors in abdominal aortic aneurisms. It was shown that the aortic expansion of mice (with aneurysm induced by elastase perfusion) in case of Nec-1s was significantly smaller than in control (64.12% vs. 172.80%; *p* < 0.05). In case of Nec-1 the aortic diameter was smaller but the difference was not statistically significant (*p* = 0.1). It was concluded that inhibition of necroptosis with Nec-1s stabilizes pre-existing aneurysms by promoting connective tissue repair and diminishing inflammation [12]. Thus, of all necrostatins, Nec-1s can be considered to be the most preferred RIP1 inhibitor. Unfortunately, not enough information is available about its fate in the organism (e.g., pharmacokinetic characteristics). Therefore, the development of a suitable analytical method for the determination of Nec-1s in biological system is of a high importance.

According to our best knowledge, however, there has been only one method established very recently for the quantitative analysis of necrostatin-1 in rat plasma [13]. The analytical procedure was based on an extraction of the analyte from the sample matrix followed by a HPLC-MS/MS analysis. The extraction recoveries of Nec-1 ranged from 85.40% to 98.25%, and lower limit of quantification was 10 μg/L. The pharmacokinetic study revealed that concentration level of Nec-1 at 5 min after i.v. administration of the drug was 1733 ng/mL. It was 617 ng/mL at 1 h, and <100 ng/mL at 8 h after administration. In case of oral administration the peak concentration (648 ng/mL) was reached at 1h after administration and was undetectable after 8 h. The determination of innovative Nec-1s in biological system, however, has not been carried out so far. Therefore, this study was undertaken to optimize and validate the HPLC-DAD-Q-TOF method for the assessment of Nec-1s levels in rat plasma what is needed for designing a proper dosing regimen for in vivo studies.

## 2. Results and Discussion

The HPLC-DAD-Q-TOF method was systematically optimized (Section 2.1), validated (Section 2.2) and applied for the determination of Nec-1s levels in rat plasma (Section 2.3). The potential of the method for biomedical use is discussed and the results are documented by appropriate figures and tables.

### 2.1. Method Development

The method development included the optimization of both the sample preparation and analytical steps. In the simple sample preparation procedure, described in Section 3.3, the plasma matrix was effectively deproteinized and preconcentrated so that any risk of interferences was reduced and sensitivity of determination enhanced. The HPLC-DAD-Q-TOF method was optimized with respect to the chromatographic separation of Nec-1s from the residual matrix constituents, and, subsequently, UV and MS detection of the pre-separated Nec-1s. A UV and MS detection tandem can be a useful tool in profiling biological matrices as the resulting profiles provide complementary information to the analyzed sample. Therefore, both detection techniques were applied and evaluated for their potentialities in Nec-1s determination in plasma.

A RP-C18 analytical chromatography column (Zorbax Extend-C18) with dimensions favorable for combination with mass spectrometry (2.1 × 50 mm, 1.8 µm) was used in the development of the HPLC-DAD-Q-TOF method. The chromatographic stationary phase was selected with respect to a maximum resolution of the relatively hydrophobic analyte from the residual plasma matrix constituents. A MS-friendly mobile phase, consisting of a mixture of 0.1% formic acid in water and 0.1% formic acid in acetonitrile, provided the best mass spectrometer response for Nec-1s. In the method optimization, methanol was also tested as an organic solvent, however, acetonitrile provided a better MS detector response. Similar observations were also made with respect to mobile phase additives. Here, 0.1% formic acid was optimum compared with 0.1% acetic acid, ammonium formate with pH 3.0 (5 mM and 10 mM), and ammonium acetate with pH 4.0 (5 mM and 10 mM). Prednisone (for the chemical structure, see Figure 1) was chosen as an internal standard (IS) based on the similarity of its chromatographic properties (log *P*) to those of Nec-1s.

A subsequent optimization of the HPLC-DAD-Q-TOF method was devoted to the creation of a proper gradient enabling the highest resolution of Nec-1s from the residual plasma matrix constituents along with the lowest retention time of the analyte. For this purpose different starting compositions of mobile phase (10–40% B) and different gradients were evaluated. Based on the peak shapes, retention times of Nec-1s and IS, peak areas and heights, and analyte vs. matrix resolution, the initial composition of mobile phase set at 25% B and linear gradient to 90% B in 4 minutes were chosen as optimum. The total analysis time, including column cleaning (90% B for 1 min) and column reconditioning (2.5 min with initial mobile phase composition), was ca. 8.0 min. 

**Figure 2:**
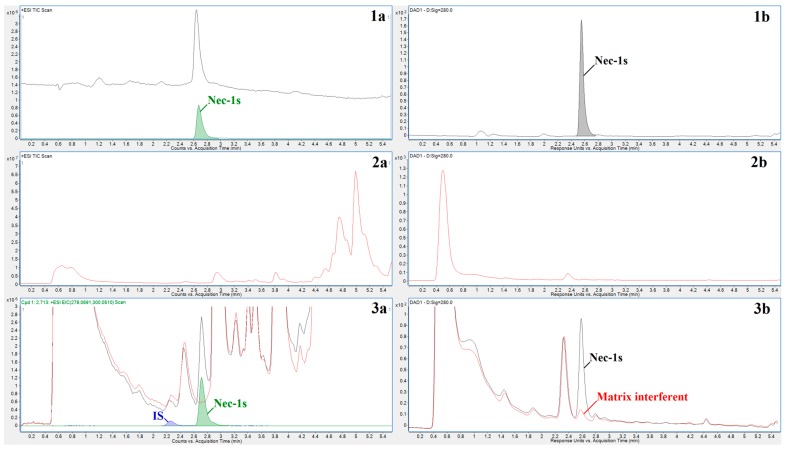
Representative chromatograms of Nec-1s in standard and plasma matrices obtained by the HPLC-DAD-Q-TOF method. MS (**a**) and DAD (**b**) profiles of: Nec-1s standard stock solution (**1**), blank plasma solution–whole chromatogram (**2**), detail of blank plasma solution (red line) overlapped with spiked plasma solution at a 2030.00 ng/mL concentration of Nec-1s (black line) (**3**). Blue and green integrated peaks in (**3a**) represent EIC of IS and Nec-1s, respectively. Optimum chromatographic and detection (UV, MS) conditions, as given in Section 3.5 and Section 3.6, respectively, were used to obtain the present profiles.

The optimized chromatographic conditions resulted in short retention times of Nec-1s and IS (˂3 min), and acceptable peak shapes (see Figure 2). The hyphenated LC-MS method also provided other key benefits such as high sensitivity of Nec-1s determination (see Section 2.2), and no significant/detectable interferences at the elution position of Nec-1s originating from the plasma matrix (see traces 3a in Figure 2). Detection of the analyte with very high resolution of its mass due to Q-TOF (see Section 3.5) was a prerequisite for avoiding any matrix interference and producing EIC spectra of the analyte with no overlaps (see e.g., panel 3a in Figure 2). On the other hand, in case of the DAD detection (evaluated at a Nec-1s absorption maximum) an interfering signal originating from the residual matrix significantly increased the Nec-1s peak area (see traces 3b in Figure 2). This interference could not be removed even if using different sample preparation procedure (e.g., extraction of Nec-1s with 3:7 hexane-ethyl acetate, unpublished results). Although, due to this interference, the LC-DAD approach was not suitable for the quantitation of Nec-1s at the concentration levels required in the present study, DAD still provided useful information about UV-absorbing compounds appearing in the recorded profiles. In our case, DAD can control absorbing peaks in the elution position of Nec-1s that are not detectable by MS (compare TIC and DAD profiles in panels 3a and 3b of Figure 2).

In the next step of the method development the operating parameters of the mass spectrometer (Q-TOF) were optimized in order to obtain maximum ionization and response of the Nec-1s. The operating parameters that were optimized included: Drying gas temperature (evaluated in the range of 200–360 °C), drying gas flow (4–12 L/min), nebulizing gas pressure (10–60 psi), capillary voltage (2000–6000 V), fragmentor voltage (50–250 V), and skimmer voltage (20–120 V).

**Figure 3:**
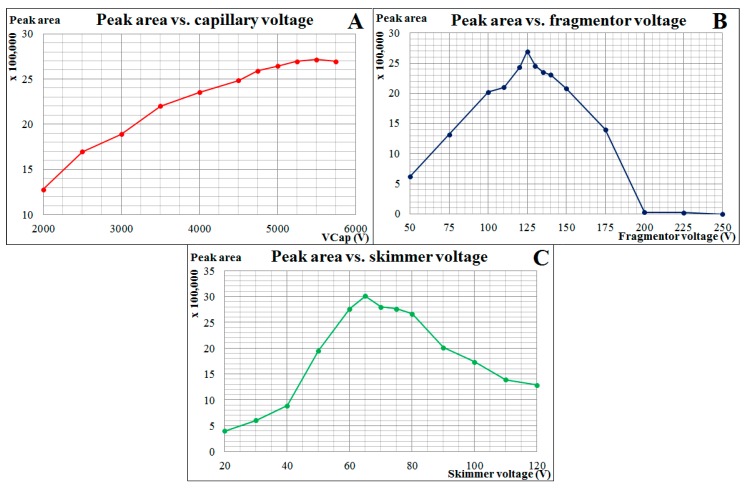
Dependences of Nec-1s detection response on selected MS parameters. FIA experiments were based on direct injection of 1 µL of Nec-1s stock solution into the Q-TOF apparatus. Diagrams represent following tested parameters: capillary voltage (**A**); fragmentor voltage (**B**); and skimmer voltage (**C**). Peak areas of Nec-1s were evaluated using its EIC profiles. For other Q-TOF parameters and *m*/*z* of the evaluated analyte adducts, see Section 3.5.

An increase of the drying gas temperature, drying gas flow, and nebulizing gas pressure resulted in an increase of the Nec-1s response (data not shown). Therefore, the maximum possible values (as limited by MS apparatus) were set for these parameters (temp. 360 °C; flow 12 L/min; pressure 60 psi). In case of the capillary voltage, fragmentor voltage, and skimmer voltage, the optimum values were chosen based on the highest peak areas obtained during a flow-injection analysis (FIA) of the Nec-1s stock solution (1 µL was injected directly into the MS apparatus). Corresponding dependences are illustrated in Figure 3. Their maxima indicated optimum values for the studied parameters, i.e. 5500 V for capillary voltage; 125 V for fragmentor voltage; 65 V for skimmer voltage.

### 2.2. Method Validation

The developed HPLC-Q-TOF method with optimized parameters was validated according to the FDA guidelines for bioanalytical methods [14]. Selectivity of the method was evaluated by comparing HPLC-Q-TOF profiles of the blank plasma (prepared as an equimolar mixture from 6 rats) and the same plasma spiked with the reference standard of Nec-1s (see diagram 3a in Figure 2). No interfering peak in the elution position of Nec-1s was observed in the profile of blank plasma. This finding confirmed an acceptable selectivity of the optimized method for monitoring Nec-1s in plasma samples.

Calibration parameters, LLOQ, and retention time of the analyte along with corresponding precision of its determination are listed in Table 1. The Nec-1s and IS peak area ratios were used for construction of the calibration line, what effectively minimized influence of fluctuations of some instrumental parameters (e.g., injection volume, column pressure, MS detection response) so that the measurements (elution times, peak areas) were characterized by a high precision (i.e., lower RSD values). The method exhibited excellent linearity in the interval of two decadic orders. The LLOQ value obtained using the Q-TOF detector (50-times higher in comparison to DAD) is favorable for the determination of Nec-1s on the concentration levels expected after the administration of a therapeutic dose of the drug to rats. Moreover, the method sensitivity can be further increased (at least in one order) when replacing Q-TOF with more sensitive QqQ.

The intra-day and inter-day precisions and accuracies of the developed method obtained during analysis of the quality control (QC) samples on three concentration levels (low, medium, high) are presented in Table 2. Each QC sample was analyzed 5-times on three consecutive days. The obtained results clearly demonstrated excellent precision (expressed as relative standard deviations, RSD) and accuracy (expressed as relative errors, RE) of the HPLC-Q-TOF method, easily accomplishing the FDA criteria recommended for biomedical analysis.

The calibration curve was obtained from the analysis of calibration solutions spiked in rat plasma (matrix) and, therefore, the matrix effects and extraction recoveries were accounted for. Despite that, for the illustration, the extraction recoveries and matrix effects were also evaluated on three concentration levels-20.30 ng/mL (low); 203.00 ng/mL (medium); 2030.00 ng/mL (high). For the preparation of solutions used for this purpose please refer to Section 3.4. The results from the evaluation of extraction recoveries and matrix effects are presented in Table 3. The data, ranging in the interval of 89.82–110.42% (SD ≤ 1.43) for the extraction recoveries and 90.75–109.94% (SD ≤ 3.60) for the matrix effects, are fully acceptable for bioanalytical method.

It can be summarized that the performance parameters of the developed HPLC-Q-TOF method, including selectivity, linear range, linearity, sensitivity, accuracy, and precision, are favorable for its biomedical application such as determination of Nec-1s levels in rat plasma (see Section 2.3).

### 2.3. Biomedical Application

Nec-1s has not been studied in clinical trials so far; only in vitro and ex vivo animal studies have been performed with this agent [10,15,16,17]. Likewise, according to our best knowledge there are no in vivo animal studies investigating the anti-necroptotic effects of Nec-1s. 

The developed and validated HPLC-Q-TOF method was applied for the determination of real plasma concentrations of Nec-1s in 5 male Wistar rats after i.v. administration of a bolus dose of Nec-1s (0.37 mg/kg). The samples taken from each subject were prepared and analyzed in triplicate. The experimentally found individual rat plasma levels of Nec-1s are summarized in Table 4. The obtained results clearly demonstrated a suitability of the HPLC-Q-TOF method to monitor trace Nec-1s levels (nM) with a high reproducibility (RSD < 2%). The measured data were consistent to each other with plasma concentrations in the range of 319.81–466.52 nM, and mean value of 377.26 nM. The differences in the found concentrations can be explained by different metabolism in the studied animals. 

When considering the concentration levels found in the pharmacokinetic study of Nec-1 (see the Introduction section) [13], the developed HPLC-Q-TOF method seems to be enough sensitive (see Section 2.2 Method validation) and, by that, easily applicable also for similar pharmacological and clinical studies of Nec-1s (even if Nec-1s exhibits 2-times higher inhibition activity than Nec-1). More specifically, the method could be useful for discovery of relationships and correlations between plasma concentrations of Nec-1s and its selected pharmacological effects (e.g., influence of the contractile and electrical activity of the heart as described in [11], or stabilization of pre-existing aneurisms [12]). Such knowledge could lead to further therapy optimization. 

## 3. Materials and Methods 

### 3.1. Chemicals and Solutions

The reference standard of Nec-1s was obtained from Merck (Darmstadt, Germany). The reference standard of prednisone (≤98%; used as internal standard, IS) was obtained from Sigma-Aldrich (Steinheim, Germany). Acetonitrile, methanol and water (all LC-MS grade) were obtained from VWR International GmbH (Vienna, Austria). Formic acid (for mass spectrometry, ~98%) was obtained from Fluka Chemie GmbH (Buchs, Switzerland).

A stock solution of Nec-1s reference standard was prepared at the concentration of 14.5 µg/mL by dissolving the calculated amount of the reference standard in 50% methanol (*v*/*v*). The stock solution of prednisone reference standard (IS) was prepared at the concentration of 50 µg/mL in the same manner. As recommended by the FDA guidelines [14], separate stock solutions were prepared for the QC samples on one side, and for the calibration solutions on the other side. Calibration solutions, QC samples, and all other solutions used in this work were prepared by a proper dilution of the stock solutions. Both Nec-1s and IS stock solutions were stored at −20 °C until use.

### 3.2. Plasma Sample Collection 

A total of 11 male Wistar rats (250–300 g body weight) were housed under standard conditions with a controlled light/dark cycle (12/12 h) and temperature (22 °C ± 2 °C), and fed a standard diet and tap water ad libitum. The anesthetized animals (natrium pentobarbital 60 mg/kg, i.p.) were randomized in two groups to receive either vehicle (C, n = 6) or Nec-1s (N, n = 5) and blood was collected from the abdominal aorta 50-min after drug application (a bolus dose; 0.37 mg/kg, i.v.; diluted in 20% *v*/*v* DMSO) into EDTA-containing syringes (S-Monovette, Starstedt, Germany). To obtain plasma, the blood was centrifuged at 4 °C for 15 min at 4000× *g*. The aliquots of plasma were stored at −80 °C until further procedure. The dosage of Nec-1s, with respect to its IC_50_ (210 nM), was chosen to get an equipotent dose to the one used in our previous in vivo experiment [11].

### 3.3. Sample Preparation

An aliquot of plasma was thawed at laboratory temperature. Afterwards, the rat plasma (180 µL) was mixed with the internal standard solution (20 µL, 1.25 µg/mL of prednisone in 50% (*v*/*v*) methanol). The mixture was briefly vortexed and 800 µL of acetonitrile was added to the mixture, which resulted in protein precipitation. The precipitated sample was vortexed for 5 min (2400 rpm) and then centrifuged for 10 min (15000× *g*; 4 °C). After the centrifugation, 800 µL of supernatant was transferred into another tube and evaporated to dryness under a gentle stream of nitrogen at ambient temperature. The residuals were reconstituted with 100 µL of mobile phase (25:75 mixture of acetonitrile and water containing 0.1% formic acid) and an aliquot of the solution (5 µL) was used for the HPLC-DAD-Q-TOF analysis.

### 3.4. Method Validation

The method’s selectivity, linear range, linearity, lower limit of quantification, accuracy, precision, matrix effect, and extraction recovery were evaluated according to the guidelines for bioanalytical methods developed by the US Food and Drug Administration (FDA) authority [14].

The calibration solutions used during the validation of the method were prepared at 6 different concentration levels of Nec-1s (20.30; 50.75; 101.50; 203.00; 507.50 and 2030.00 ng/mL) by spiking the equimolar mixture of blank plasmas obtained from six rats (receiving only vehicle C). The preparation procedure of the calibration solutions (spiked plasma) was the same as for the sample (see Section 3.3). For this purpose, the properly diluted solutions of Nec-1s reference standard were mixed with both the internal standard solution and blank plasma (180 µL of plasma, 10 µL of Nec-1s, 10 µL of 2.5 µg/mL IS). Each calibration solution was analyzed in triplicate.

The calibration curve was prepared using the peak area ratios of Nec-1s to IS (obtained from the analysis of the calibration solutions with known concentrations) and applying the weighted linear regression (MS Excel 2010). The LLOQ is represented by the lowest concentration in the calibration curve for which the response is at least five times the response compared to blank and the accuracy is within ±20% of nominal value and precision is not greater than 20% (according to [14]).

QC samples were prepared at three concentration levels (20.30 (low); 203.00 (medium) and 2030.00 (high) ng/mL) and each was measured five times for three consecutive days in order to evaluate intra-day and inter-day accuracy and precision of the method.

Matrix effects were evaluated by comparing the peak areas obtained by the analysis of plasma samples spiked post-extraction with the peak areas obtained by the analysis of reference standard solution diluted with mobile phase. Extraction recovery was evaluated by comparing the peak areas obtained by the analysis of plasma samples spiked pre-extraction and plasma samples spiked post-extraction. Both the matrix effects and extraction recovery were evaluated at three concentration levels (20.30; 203.00, and 2030.00 ng/mL).

### 3.5. LC-DAD/MS Apparatus

**Figure 4:**
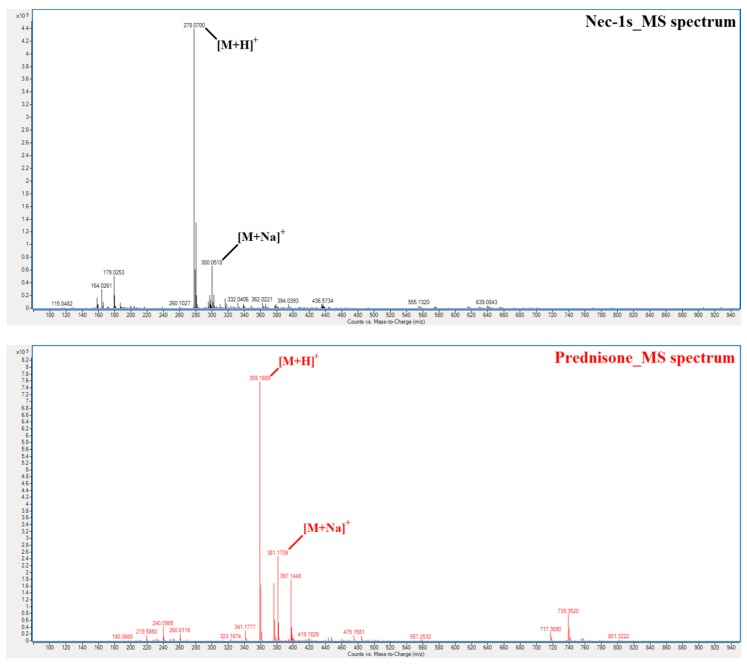
MS spectra of Nec-1s and prednisone. FIA analysis of Nec-1s (**upper panel**) and prednisone (**lower panel**) stock solutions showing experimental masses for their H^+^ and Na^+^ adducts.

All of the analyses were carried out on the chromatographic apparatus consisting of LC Agilent Infinity System (Agilent Technologies, Santa Clara, CA, USA) equipped with a gradient pump (1290 Bin Pump VL), an automatic injector (1260 HiPals), and column thermostat (1290 TCC; set at 4 °C). The LC system was hyphenated with a photodiode array detector (Infinity 1290 DAD), and quadrupole time-of-flight (Q-TOF) mass spectrometer (Agilent 6520 Accurate-Mass Q-TOF LC/MS) equipped with the electrospray ionization (ESI) source operated in a positive ionization mode. A computer with a MassHunter software (version MassHunter Workstation B.05.01, Agilent Technologies) was used to control the apparatus as well as acquire and process the data. The Q-TOF mass spectrometer was operated under following parameters: drying gas temperature 360 °C, drying gas flow 12 L/min, nebulizing gas pressure 60 psi, capillary voltage 5500 V, fragmentor voltage 125 V, skimmer voltage 65 V, OctopoleRFPeak 550 V, and collision gas N_2_. The processing of the obtained chromatograms (TIC profiles) resulted in the extracted ion chromatograms (EIC) for Nec-1s and prednisone. In the processing the exact (theoretical) masses of their [M + H]^+^ (278.0691 for Nec-1s; 359.1853 for prednisone), and [M + Na]^+^ (300.0510 for Nec-1s; 381.1672 for prednisone) adducts were used. For the MS spectra obtained by the analysis of the stock solutions of Nec-1s and prednisone see Figure 4. The theoretical and experimentally found masses of the Nec-1s and prednisone adducts were in a very good mutual agreement so that Q-TOF was suitable for an unambiguous confirmation of the identity of the analyzed compounds in the samples. The EIC were extracted with *m*/*z* expansion of ±10 ppm. In case of DAD detector, the peak areas of the analyte and IS were evaluated at the wavelength of 280 nm.

### 3.6. Chromatographic Conditions

The chromatographic separation of the Nec-1s was achieved by using RP-C18 chromatographic column–Zorbax Extend-C18, 2.1 × 50 mm, 1.8 µm obtained from Agilent Technologies. A gradient elution of mobile phases consisting of 0.1% formic acid in LC-MS water (A) and 0.1% formic acid in acetonitrile (B) was used. The gradient elution was set as follows: starting composition of the mobile phases was 25% B, which was held for 0.5 min, then increased to 90% B within next 4.0 min, and held at 90% B for another 1.0 min. The column was reconditioned for 2.5 min with starting composition of the mobile phases. The flow rate of mobile phases was at 300 µL/min during the whole analysis. The column temperature was maintained at 40 °C.

## 4. Conclusions

These experimental results clearly demonstrated a suitability of the developed and successfully validated HPLC-DAD-Q-TOF method with a simple sample preparation for a highly reliable determination of trace Nec-1s concentration levels in rat plasma matrices. The presented biomedical application provided reproducible and consistent data for rat plasma levels of Nec-1s, given intravenously as a bolus dose, enabling us to recognize differences in investigated model biological systems (e.g., metabolic activity). The proposed analytical protocol, meeting the FDA criteria, is favorable for routine use, with application areas including therapeutic drug monitoring (for optimization of therapeutic dose) or pharmacokinetic studies of this innovative highly effective drug necrostatin. Further both analytical and pharmacological studies are required to evaluate Nec-1s with respect to its organ selectivity and efficacy in conditions characterized by necroptotic damage. 

## Figures and Tables

**Table 1:** Calibration and selected performance parameters of developed HPLC-Q-TOF method for Nec-1s in rat plasma.

RT (min)	RSD_RT_ (%)	Linear Range (ng/mL)	Linearity r^2^	LLOQ (ng/mL)	Regression Equation			
					b	SD ^b^	a	SD ^a^
2.72	0.57	20.3–2030.0	0.9991	20.3	4.062	0.028	−0.014	0.023

RT—retention time; RSD—relative standard deviation; b—slope; a—intercept; SD—standard deviation; r^2^—coefficient of determination; LLOQ—lower limit of quantification.

**Table 2:** Precision and accuracy of Nec-1s in rat plasma (analysis of QC samples).

QC (ng/mL)	Intra-Day, n = 5		Inter-Day, n = 15	
	Precision (RSD %)	Accuracy (RE %)	Precision (RSD %)	Accuracy (RE %)
20.30	1.78	106.76	4.59	107.89
203.00	1.28	108.52	1.97	106.95
2030.00	0.97	99.07	1.83	97.45

**Table 3:** Extraction recoveries and matrix effects of Nec-1s in rat plasma (mean ± SD, n = 5).

QC (ng/mL)	Extraction Recoveries (%)	Matrix Effects (%)
20.30	89.82 ± 1.43	109.94 ± 3.60
203.00	102.58 ± 1.36	90.75 ± 0.43
2030.00	110.40 ± 0.65	97.20 ± 0.40

**Table 4:** Plasma concentrations of Nec-1s found in rats 50 min after i.v. bolus dose (0.37 mg/kg) administration.

Sample	Plasma Concentration (ng/mL)	Plasma Concentration (nM)	RSD% (n = 3)
N1	103.99	374.44	0.74
N2	90.93	327.43	1.37
N3	110.55	398.08	0.81
N4	88.81	319.81	0.54
N5	129.56	466.52	0.41
